# MND1 reduces breast cancer chemosensitivity by promoting RAD51-mediated homologous recombination repair

**DOI:** 10.1038/s41419-026-08912-w

**Published:** 2026-06-03

**Authors:** Hao-Ran Yue, Zhi-Hao Yu, Hong-Meng Zhao, Lin-Yue Hai, Wen-Bo Liu, Xiao-Feng Liu, Zhao-Hui Chen, Yue Yu, Jie Ge, Xin Wang

**Affiliations:** 1 The First Department of Breast Cancer, Tianjin Medical University Cancer Institute and Hospital, National Clinical Research Center for Cancer, Tianjin, China; 2 Key Laboratory of Cancer Prevention and Therapy, Tianjin, China; 3 Tianjin’s Clinical Research Center for Cancer, Tianjin, China; 4 Key Laboratory of Breast Cancer Prevention and Therapy, Tianjin Medical University, Ministry of Education, Tianjin, China

**Keywords:** Breast cancer, Homologous recombination

## Abstract

Breast cancer remains a major global health challenge for women. Chemotherapy resistance is a key driver of breast cancer recurrence and metastasis. Although meiotic nuclear division 1 (MND1) has been characterized as an oncogenic factor involved in mitotic progression and homologous recombination (HR), the precise molecular mechanisms underlying these MND1-mediated processes remain unclear. The expression of MND1 in breast cancer cell lines was evaluated by RT-qPCR and western blot. The functional effects of MND1 were assessed through a series of in vitro and in vivo assays, including cell proliferation assays, determination of resistance curves, DNA damage detection and flow cytometry. Protein interactions among MND1, RAD51, and USP5 were determined by co-immunoprecipitation assays. Transcriptional regulation of MND1 by E2F1 and promoter methylation status were analyzed using luciferase reporter assays and bisulfite sequencing PCR, respectively. We demonstrated that MND1 is upregulated in breast cancer and associated with cancer progression and cisplatin resistance. Mechanistically, MND1 promotes HR repair by recruiting USP5 to deubiquitinate and stabilize RAD51. Furthermore, E2F1 induces promoter hypomethylation and transcriptionally activates MND1 by binding to its promoter. Our findings establish a role for MND1 in regulating chemosensitivity and provide new insights into relevant gene interaction networks. The identified E2F1/MND1/USP5/RAD51 feedback loop underscores the potential of MND1 as a therapeutic target for breast cancer.

## Introduction

Breast cancer remains a major global health burden and the leading cause of morbidity and mortality in women worldwide [1, 2]. Despite significant advances in breast cancer therapy, chemotherapy resistance remains a major clinical obstacle that often leads to treatment failure and disease relapse [3]. The mechanisms underlying chemotherapy resistance in breast cancer are multifactorial, involving both intracellular and extracellular factors. Intracellular mechanisms include altered drug metabolism, enhanced drug efflux, and modifications of drug targets, whereas extracellular mechanisms involve complex interactions between tumor cells and their microenvironment [4–6]. Nevertheless, the underlying oncogenic pathways and novel candidate driver genes in breast cancer require further elucidation.

The meiotic nuclear division 1 (MND1) gene, located on chromosome 14q24.3, is essential for meiotic recombination and DNA repair, specifically through its role in mediating homologous recombination (HR) [7, 8]. MND1 functions as a heterodimer with PSMC3IP. This heterodimer is indispensable for promoting D-loop formation during meiotic HR, a process critical for repairing DNA double-strand breaks (DSBs) [9]. MND1 is aberrantly expressed in several cancer types. For example, in prostate cancer, MND1 upregulation accelerates cell cycle progression by activating the CCNB1/p53 axis, thereby reinforcing its oncogenic potential [10]. In lung adenocarcinoma, MND1 participates in a positive feedback loop with E2F1 and KLF6, thereby promoting tumor progression and therapy resistance through cell cycle dysregulation [11]. Furthermore, MND1 expression correlates with the abundance of tumor-infiltrating immune cells and the expression of immune checkpoint markers, such as PD-L1, PD-1 and CTLA-4, suggesting a role in modulating the tumor immune microenvironment [12, 13]. However, the specific molecular mechanisms and functional roles of MND1 in breast cancer pathogenesis remain to be elucidated.

DNA damage plays a pivotal role in both oncogenesis and cancer therapy [14, 15]. The ataxia-telangiectasia mutated (ATM) kinase is a central regulator of the DNA damage response (DDR) and is primarily activated by DNA double-strand breaks (DSBs) [16]. Upon activation, ATM orchestrates a signaling cascade that phosphorylates key substrates including p53, CHK2 and H2AX, thereby facilitating cell cycle arrest, DNA damage repair and apoptosis [17]. Given its essential function in maintaining genomic stability, aberrant ATM activity is tightly associated with tumor onset and malignant progression. For instance, SERPINE2 silencing inhibits phosphorylated ATM accumulation and suppresses RAD51-mediated DNA repair. Conversely, RAD51 overexpression can restore DNA damage repair capacity and radioresistance in lung cancer cells [18]. Additionally, ATM promotes irradiation-induced autophagy in H1299 lung cancer cells *via* the MAPK14/mTOR pathways and Beclin1/PI3KIII complexes [19]. Existing studies reveal that the USP10/XAB2/ANXA2 cascade enhances DNA damage repair, thereby contributing to oxaliplatin resistance in colorectal cancer. In breast cancer, YTHDF1 modulates E2F8 expression to accelerate DNA damage repair, which desensitizes tumor cells to adriamycin, cisplatin and olaparib [20, 21]. Therefore, a detailed understanding of the molecular mechanisms governing DNA damage repair is crucial for developing improved breast cancer therapies.

This study demonstrates that MND1 is a direct transcriptional target of E2F1 and physically interacts with RAD51 in chemotherapy-resistant breast cancer cells. Furthermore, MND1 recruits USP5 to deubiquitinate RAD51, thereby stabilizing it by preventing its degradation. Chemotherapy-induced DNA damage is sensed by ATM, which triggers RAD51-mediated homologous recombination repair. Subsequently, E2F1 is reactivated as a downstream effector. Collectively, our findings reveal a MND1/USP5/RAD51/E2F1 regulatory feedback loop that critically contributes to breast cancer pathogenesis.

## Materials and methods

### Cell culture, plasmids, and transfection

MDA-MB-231 (RRID: CVCL_0062), CAL-51 (RRID: CVCL_1110), T47D (RRID: CVCL_0553), MCF7 (RRID: CVCL_0031), MCF10A (RRID: CVCL_0598), HEK293T (RRID: CVCL_0063), BT-549 (RRID: CVCL_1092) and U2OS (RRID: CVCL_0042) were obtained from the Cell Bank of the Chinese Academy of Sciences (Shanghai, China). MCF10A cells were maintained in MCF-10A specialized medium (Procell, China). CAL-51, BT-549, and U2OS cells were cultured in 1640 medium (Gibco, USA), whereas MCF7, T47D, MDA-MB-231, and HEK 293T cells were cultured in DMEM (Gibco, USA). Mycoplasma contamination was excluded on arrival and every 3 months thereafter using a PCR-based assay. Detailed information on plasmids, PCR primers, shRNA sequences and transfection reagents is provided in Data S1 and Table S1.

To establish Cisplatin-resistant MDA-MB-231 cell line (MDA-MB-231/DDP), we employed a long-term dose-escalation method by culturing parental MDA-MB-231 cells in media containing cisplatin (D8810, Solarbio). The initial drug concentration was set at IC_50_ of the parental cells, and the medium was changed every 2 days. The drug concentration was increased incrementally by 50–100 ng/mL per step when the cells exhibited stable growth without signs of cytotoxicity. The maximum concentrations were 2 μg/mL, with resistant cells maintained and passaged at these concentrations. Then IC_50_ value of MDA-MB-231/DDP was determined and the drug resistance index (RI) was calculated. If cell growth became unstable, the drug concentration was reduced, and escalation was resumed once stability was restored. The development of resistant cell lines required 6 months for MDA-MB-231/DDP cells.

### Clinical samples

Breast cancer tissue specimens and matched adjacent normal tissues were obtained from Tianjin Medical University Cancer Institute and Hospital (TMUCIH). All tumor samples were obtained from patients newly diagnosed with breast cancer and who had received no therapy before sample collection. The tissues were stored at −80 °C for the following analysis. All procedures were approved by the Institutional Ethics Committee of TMUCIH and were performed in accordance with the ethical standards. Written informed consent was obtained from all participants.

### Immunohistochemistry

Deparaffinized tissue sections underwent heat-induced antigen retrieval in 0.01 M sodium citrate buffer at 98 °C for 18 min. 3% H_2_O_2_ was used to inhibit endogenous peroxidase activity. Sections were then blocked with 5% bovine serum albumin (BSA) for 1 h at room temperature and incubated with primary antibodies overnight at 4 °C. After washing with PBS, sections were incubated with HRP-conjugated secondary antibodies for 1 h at room temperature. Signals were visualized using 3,3’-diaminobenzidine (DAB), counterstained with hematoxylin, differentiated in acid alcohol, and mounted with neutral gum.

### Western blot and immunofluorescence

Cellular lysates were separated by SDS-PAGE, transferred to membranes, and immunoblotted with specific primary antibodies. Protein bands were visualized using an ECL reagent (Millipore, Bedford, MA, USA). Protein band intensity was quantified by densitometry using ImageJ software and normalized to β-actin.

Cells were seeded at a density of 3 × 10⁴ cells per well on glass coverslips for immunofluorescence analysis. Cells were then washed, fixed, and permeabilized. After incubation with primary antibodies overnight at 4 °C, cells were incubated with FITC- or TRITC-conjugated secondary antibodies for 1 h at room temperature. DAPI was used to counterstain the cells, and a Zeiss fluorescence microscope was used to take pictures. Details of antibodies and reagents applied in these experiments are provided in Data S1.

### RNA extraction and reverse transcription quantitative polymerase chain reaction (RT-qPCR)

Total RNA was extracted from cultured cells using the SparJade RNA Kit according to the manufacturer’s instructions. cDNA was reverse-transcribed from total RNA with 5 × PrimeScript RT Master Mix (TransGen Biotech). RT-qPCR was performed using 2 × SYBR Green Mix (TransGen Biotech) on a Bio-Rad quantitative detection system. All primer sequences used in this study are listed in Table S2.

### Proliferation and invasion assay

Cell proliferation was assessed via colony formation, EdU (5-ethynyl-2’-deoxyuridine), and CCK-8 (Cell Counting Kit-8) assays. Cell migration and invasion were evaluated using wound-healing and Transwell assays, respectively. Detailed protocols for these functional assays are provided in Data S1.

### Co-immunoprecipitation (Co-IP) and protein identification

Cells were lysed with BC100 buffer 24 h after transfection to extract ectopically expressed proteins. Whole-cell lysates were incubated with specific antibodies overnight at 4 °C, followed by immunoprecipitation with protein A/G beads or M2 agarose beads (Sigma-Aldrich). After washing three times with PBS lysis buffer, the precipitated proteins were eluted by boiling in 2 × SDS loading buffer at 100 °C for 10 min, and then analyzed by SDS-PAGE. Protein identification by mass spectrometry was performed by Novogene company (China).

### GST pull-down

To obtain fusion proteins, BL21 (DE3) cells were transformed with plasmids encoding GST-RAD51 and His-USP5. GST and GST-RAD51 fusion proteins (100 µg each) were immobilized on 50 µL of glutathione agarose beads by incubation with gentle agitation for 1 h at 4 °C. Following three washes with PBST, 100 µg of His-USP5 fusion protein was added to the immobilized GST-RAD51/GST complexes. The bound proteins were eluted with elution buffer (10 mM glutathione in PBS, pH 8.0) and analyzed by western blot.

### Flow cytometry analysis

For cell cycle analysis, cells were harvested, fixed in 70% ice-cold ethanol overnight at 4 °C, and then stained with propidium iodide (PI, BD Biosciences) solution. For apoptosis analysis, cells were resuspended in 1× binding buffer and stained with 5 μL APC Annexin V and 5 μL PI for 15 min at room temperature in the dark according to the manufacturer’s guidelines.

### Comet assay

Following chemotherapy treatment, cells were harvested, reconstituted into single-cell suspensions, and combined with 1% agarose at a density of 1 × 10⁵ cells/mL prior to being placed onto slides. After solidification, the slides were immersed in lysis buffer (2% sarkosyl, 0.5 M Na₂EDTA, and 0.5 mg/mL proteinase K) and incubated overnight at 37 °C in the dark. Electrophoresis was performed in freshly prepared N₂ buffer (90 mM Tris, 90 mM boric acid, and 2 mM Na₂EDTA) for 25 min at 120 V. Following electrophoresis, the slides were neutralized, stained with PI (2.5 mg/mL) for 30 min at room temperature, and visualized under a fluorescence microscope.

### Chromatin immunoprecipitation (ChIP) and luciferase reporter assays

ChIP was performed using a commercial kit (Millipore, USA) according to the manufacturer’s instructions. Briefly, cells were cross-linked with 1% paraformaldehyde, lysed in a nuclear lysis buffer containing protease inhibitors, and the chromatin was sheared by sonication. The lysates were incubated with specific antibody overnight at 4 °C, followed by immunoprecipitation with protein A/G agarose beads. The antibody-bound chromatin complexes were collected and washed, after which the protein-DNA cross-links were reversed. The purified DNA was analyzed by PCR using the primers listed in Table S3.

For luciferase reporter assay, the MND1 promoter regions were cloned into the pGL3-basic firefly luciferase vector. Breast cancer cells were then co-transfected with the constructed pGL3-promoter plasmids and the pRL-TK plasmid (Promega), which expresses Renilla luciferase and serves as an internal control for normalization. Firefly and Renilla luciferase activities were measured 24–48 h post-transfection using the Dual-Luciferase Reporter Assay System (TransGen, China) following the manufacturer’s protocol.

### DNA isolation and bisulfite sequencing PCR (BSP)

Genomic DNA was extracted from cells and tissues using a commercial kit (Thermo Scientific, USA). The extracted DNA was then treated with the EpiTect bisulfite kit (Qiagen, Germany) to convert unmethylated cytosines to uracil. The bisulfite-converted PCR products were purified using the EasyPure Quick Gel Extraction Kit (TransGen, China). The purified PCR products were cloned into a vector, and ten randomly selected clones from each ligation were sequenced. The methylation status of individual CpG sites was determined by comparing the sequencing results to the original genomic sequence; cytosines that remained as C (rather than converting to T) were scored as methylated. The primers used for BSP are listed in Table S3.

### Xenograft

Stable MND1-overexpressing MDA-MB-231 cells and control cells (3 × 10^6^) were generated and mixed with 100 μg of Matrigel (BD Biosciences, San Diego, CA, USA). The cell suspension was then orthotopically injected into the mammary fat pads of 5-week-old female severe combined immunodeficiency (SCID) mice. Tumor growth was monitored twice a week by measuring tumor dimensions with a digital caliper. Tumor volume was calculated using the formula: volume = (length × width^2^)/2. Metastatic burden was assessed at the endpoint (week 5) by bioluminescence imaging using a Xenogen IVIS 200 Imaging System (Caliper Life Sciences, Hopkinton, MA, USA). All mice were euthanized at the 5-week endpoint, and primary tumors were excised and weighed. Final tumor volumes were recorded prior to excision. All animal experiments were approved by the Animal Ethics Committee of TMUCIH. All procedures were performed in strict accordance with the institutional guidelines for the care and use of laboratory animals and the national animal welfare regulations.

### RNA-seq analysis

Total RNA was extracted from cells using TRIzol reagent (Invitrogen) according to the manufacturer’s instructions. RNA integrity was assessed using an Agilent 2100 Bioanalyzer (Agilent Technologies). Libraries were prepared using the NEBNext Ultra RNA Library Prep Kit (NEB) and sequenced on an Illumina NovaSeq 6000 platform (Illumina) with 150 bp paired-end reads. Raw sequencing reads were processed to remove adapter sequences and low-quality bases using Trimmomatic (v0.39). Clean reads were then aligned to the human reference genome (GRCh38) using RSEM (v1.3.3), and gene-level expression counts were obtained. Differentially expressed genes (DEGs) were identified using the DESeq2 package (v1.42.0) in R (v4.2.0). Genes with an absolute log₂ fold change (|log_2_FC|) ≥ 1 and a Benjamini-Hochberg (BH) adjusted *p*-value (false discovery rate, FDR) < 0.05 were considered significantly differentially expressed.

### Statistical analysis

All data were confirmed to follow a normal distribution using the Shapiro-Wilk test. Data were presented as mean ± SD from at least three independent experiments. Statistical comparisons between two groups were performed using two-tailed Student’s *t*-test, and among multiple groups using one-way ANOVA followed by Tukey’s post hoc test. *p* < 0.05 was considered statistically significant. Data were visualized using GraphPad Prism 8.0.

### Ethical approval and consent to participate

This study and included experimental procedures were approved by the Animal Ethical and Welfare Committee of Tianjin Medical University Cancer Institute and Hospital (NSFC-AE-2024056). All participants provided their written informed consent. All processes were carried out in complete accordance with the Helsinki Declaration of 1964 and its subsequent revisions.

## Results

### MND1 is upregulated in cisplatin-resistant breast cancer

**Fig. 1:**
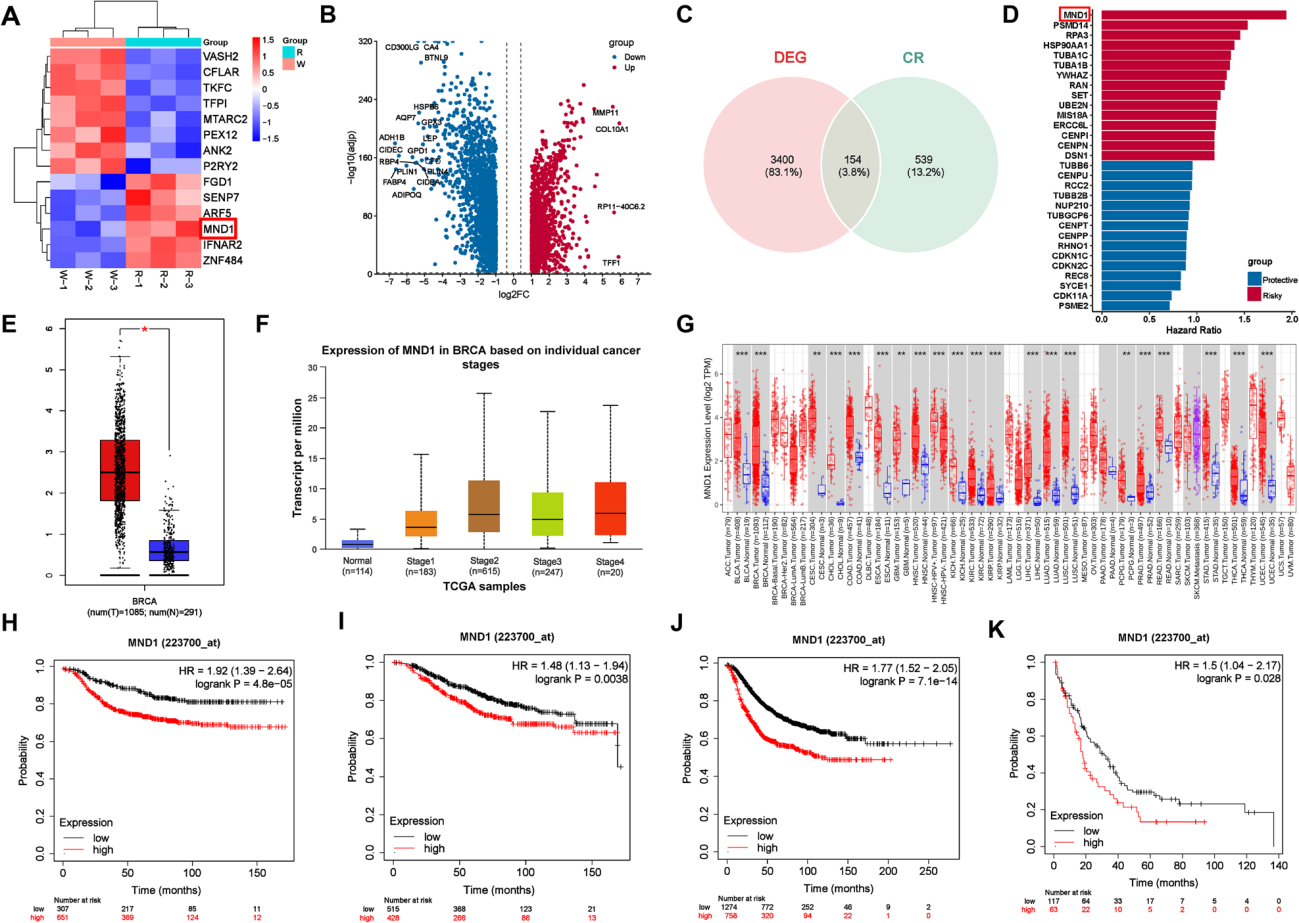
MND1 is upregulated in cisplatin-resistant breast cancer. **A** Heatmap of differentially expressed genes (DEGs) in parental versus cisplatin-resistant breast cancer cells. **B** Volcano plot of DEGs between breast cancer and normal tissues from TCGA-BRCA cohort. **C** Venn diagram identifying overlapping DEGs from (**A**) and (**B**). **D** Univariate Cox regression analysis of the overlapping genes from (**C**). **E** MND1 mRNA expression levels in breast cancer and normal tissues from the TCGA database. **F** MND1 expression levels across different clinical stages of breast cancer. **G** Pan-cancer analysis of MND1 mRNA expression in various tumor types (red) versus matched normal tissue (blue). Kaplan-Meier survival analysis comparing patients with high (red) versus low (black) MND1 expression for (**H**) distant metastasis free survival (DMFS), **I** overall survival (OS), **J** recurrence-free survival (RFS), and **K** post progression survival (PPS). *p < 0.05, **p < 0.01, ***p < 0.001.

To identify genes associated with cisplatin resistance, we performed RNA sequencing on cisplatin-resistant and parental breast cancer cell lines, which revealed a set of differentially expressed genes (Fig. 1A). Subsequently, we analyzed transcriptomic data from The Cancer Genome Atlas (TCGA)-BRCA (https://portal.gdc.cancer.gov/) cohort and identified differentially expressed genes between breast cancer tissues and adjacent normal tissues (Fig. 1B). The intersection of these two gene sets — from the cisplatin-resistant cells and the TCGA cohort—yielded 154 candidate genes (Fig. 1C). Univariate Cox regression analysis of these candidates identified the top 30 high-risk and protective genes (Fig. 1D). Using the Gene Expression Profiling Interactive Analysis (GEPIA) platform (http://gepia.cancer-pku.cn/), we confirmed that MND1 mRNA expression was significantly upregulated in breast cancer tissues compared with normal controls (Fig. 1E). Furthermore, MND1 expression increased with advancing clinical stage (Fig. 1F). Pan-cancer analysis indicated that MND1 functions as an oncogene in most cancer types (Fig. 1G). Kaplan–Meier plotter analysis (http://kmplot.com/analysis) further revealed that high MND1 expression was significantly associated with poorer clinical outcomes, including diminished distant metastasis-free survival (DMFS), overall survival (OS), recurrence-free survival (RFS), and post-progression survival (PFS) (Fig. 1H–K). Collectively, these findings establish that MND1 is upregulated in breast cancer, associated with cisplatin resistance, and correlated with unfavorable prognosis in patients with breast cancer.

### MND1 promotes breast cancer proliferation, invasion and chemoresistance

**Fig. 2:**
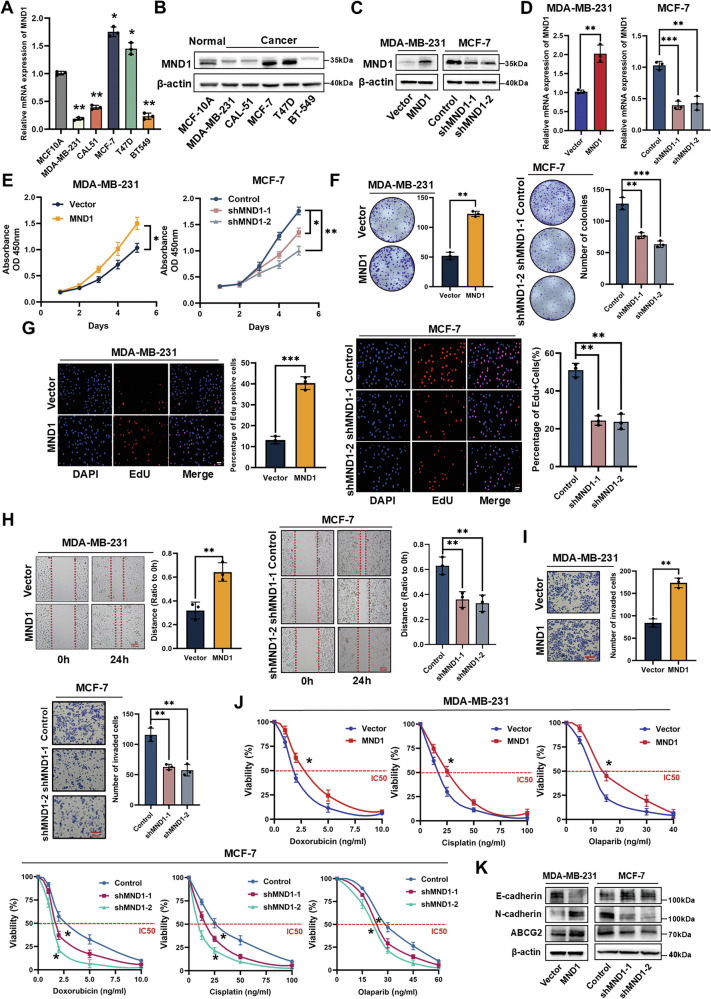
MND1 promotes breast cancer proliferation, invasion and chemoresistance. Endogenous MND1 expression was evaluated in a panel of breast cancer cell lines and the normal breast epithelial cell line MCF10A by RT-qPCR (**A**) and western blot (**B**). Stable MND1-overexpressing MDA-MB-231 (left) and MND1-depleted MCF-7 (right) cell lines were validated by western blot (**C**) and RT-qPCR (**D**). Functional consequences of MND1 modulation on cell proliferation were assessed by CCK-8 (**E**), colony formation (**F**), and EdU (**G**) assays. Effects of MND1 on cell migration and invasion were evaluated by wound healing (**H**) and Transwell (**I**) assays. **J** Dose-response curves and calculated IC₅₀ values for doxorubicin, cisplatin and olaparib in MDA-MB-231 and MCF-7 cells with modulated MND1 expression. **K** Western blot analysis of epithelial-mesenchymal transition (EMT) markers (E-cadherin, N-cadherin) and the drug efflux transporter ABCG2 in MND1-overexpressing MDA-MB-231 (left) and MND1-knockdown MCF-7 (right) cells. *p < 0.05, **p < 0.01, ***p < 0.001.

We first examined MND1 expression in a panel of breast cancer cell lines using RT-qPCR (Fig. 2A) and western blot (Fig. 2B). MND1 was significantly upregulated at both mRNA and protein levels in certain breast cancer cell lines tested (MCF-7 and T47D) compared to the normal breast epithelial cell line MCF-10A and triple-negative breast cancer (TNBC) cell lines (MDA-MB-231, CAL-51 and BT-549). We established and validated stable MND1-overexpressing MDA-MB-231 cells and MND1-depleted MCF-7 cell lines using western blot and RT-qPCR (Fig. 2C, D). Functional assays were performed to assess the impact of MND1 on cell proliferation. CCK-8, colony formation and EdU assays consistently demonstrated that MND1 overexpression significantly enhanced cell proliferation compared to control cells. Conversely, MND1 knockdown attenuated these proliferative capacities (Fig. 2E–G). The effect of MND1 on cell migration and invasion was evaluated using wound healing and Transwell assays. MND1 overexpression markedly enhanced the migration and invasion capabilities of MDA-MB-231 cells, while MND1 knockdown inhibited the migration and invasion capabilities of MCF-7 cells (Fig. 2H, I), demonstrating that MND1 promotes breast cancer cell migration and invasion. Consistent results were obtained in CAL-51 cells (Fig. S1A–G).

To assess the effect of MND1 on chemosensitivity, cells with modulated MND1 expression were treated with doxorubicin, cisplatin and olaparib for 24 h and then seeded into 96-well plates. Cell viability was measured by CCK-8 assay. The IC_50_ values for all three drugs were significantly higher in MND1-overexpressing MDA-MB-231 (Fig. 2J), CAL-51 (Fig. S1H) and MCF-7 (Fig. S1I) cells compared to the control cells. Conversely, MND1 knockdown in MCF-7 cells (Fig. 2J) and MDA-MB-231 (Fig. S1J) significantly decreased the IC_50_ values for all three drugs. Previous evidence demonstrates that cisplatin inhibits breast cancer metastasis by preventing early EMT [22]. Furthermore, ABCG2/BCRP, a drug efflux transporter, is frequently dysregulated in drug-resistant cancers [23, 24]. We observed that the expression levels of N-cadherin and ABCG2 were significantly elevated, whereas the expression level of E-cadherin was reduced in MND1-overexpressing MDA-MB-231 cells compared to control cells (Fig. 2K, left). Conversely, MND1 knockdown yielded the opposite protein expression profile (Fig. 2K, right), further supporting a positive role for MND1 in promoting EMT and chemotherapy resistance. Collectively, these results demonstrate that MND1 promotes breast cancer cell proliferation, migration, invasion and chemoresistance.

### MND1 reduces chemosensitivity by enhancing DNA homologous recombination repair

**Fig. 3:**
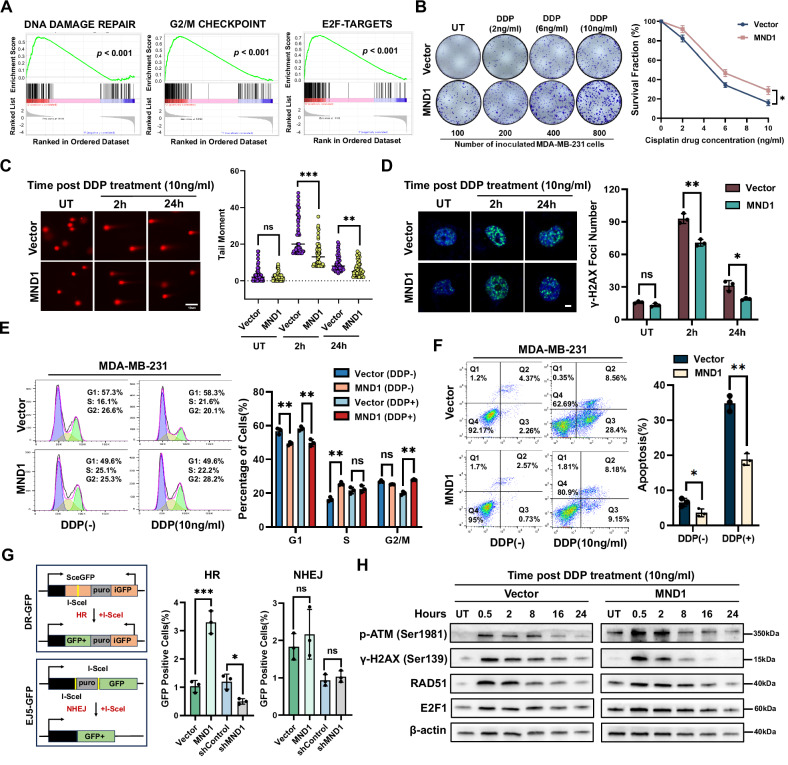
MND1 reduces chemosensitivity by enhancing DNA homologous recombination repair. **A** Gene Set Enrichment Analysis (GSEA) of RNA-seq data from MDA-MB-231 cells with modulated MND1 expression, showing enrichment for DNA damage repair, G2/M checkpoint, and E2F target gene sets. **B** Colony formation analysis of cell viability in MND1-overexpressing MDA-MB-231 and control cells treated without or with increasing concentrations of cisplatin. **C** Quantification of DNA damage (comet tail moment) by neutral comet assay at indicated time points after chemotherapy. **D** Immunofluorescence analysis of γ-H2AX foci formation and resolution over time post-chemotherapy, indicating DNA damage repair kinetics. Flow cytometry analysis of cell cycle distribution (**E**) and apoptosis (**F**) in MND1-overexpressing MDA-MB-231 and control cells with or without chemotherapy. **G** Homologous recombination (HR) and non-homologous end joining (NHEJ) repair efficiency measured by DR-GFP and EJ5-GFP reporter assays in U2OS cells. **H** Western blot analysis of key DNA damage response (phospho-ATM, γ-H2AX, RAD51) and cell cycle (E2F1) proteins in MND1-overexpressing MDA-MB-231 and control cells treated with cisplatin. *p < 0.05, **p < 0.01, ***p < 0.001.

To investigate the mechanism by which MND1 promotes chemoresistance, RNA sequencing was performed on MDA-MB-231 cells with modulated MND1 expression. Gene Set Enrichment Analysis (GSEA) revealed that MND1 overexpression was positively enriched in gene sets involved in DNA damage repair, G_2_/M checkpoint regulation, and E2F targets (Fig. 3A). Many chemotherapeutic agents exert their cytotoxic effects by inducing DNA damage [25]. We therefore assessed the functional role of MND1 in DDR to cisplatin, one of the chemotherapy drugs that can cause DNA damage, using a series of complementary assays. Colony formation assays were used to assess cell viability after cisplatin-induced DNA damage at various concentrations. The results demonstrated that the MND1-overexpressing group exhibited significantly higher cell viability than the control group as the cisplatin concentration increased (Fig. 3B). In the comet assay, DNA damage (indicated by tail length) gradually increased within the first 2 h post-treatment and was largely resolved by 24 h. At the 2 h time point, MND1-overexpressing MDA-MB-231 (Fig. 3C) and CAL-51 (Fig. S2A) cells exhibited significantly shorter comet tails than control cells, indicating less initial DNA damage or more rapid early repair. Immunofluorescence staining for γ-H2AX foci showed that while foci numbers increased in all cells after chemotherapy, MND1-overexpressing cells MDA-MB-231 (Fig. 3D) and CAL-51 (Fig. S2B) displayed more rapid foci resolution, with a significantly lower foci count at 2 h and 24 h post-chemotherapy, indicating accelerated DNA damage repair. Cell cycle analysis revealed that MND1 overexpression promoted the G1 to S phase transition in untreated cells. Following irradiation, MND1-overexpressing MDA-MB-231 (Fig. 3E) and CAL-51 (Fig. S2C) cells exhibited a more pronounced G2/M arrest, a checkpoint that allows time for DNA damage repair. As expected, cisplatin increased apoptosis. However, MND1-overexpressing MDA-MB-231 (Fig. 3F) and CAL-51 (Fig. S2D) cells were significantly protected from chemotherapy-induced apoptosis compared to controls. Previous studies have shown that cisplatin induces DNA damage primarily through intrastrand crosslinks, which are repaired via nucleotide excision repair (NER) [26]. To rule out the involvement of other common repair pathways, we examined key NER factors (XPA, XPC, and ERCC1) and key mismatch repair (MMR) factors (MLH1, MSH2, and MSH6) by western blot at 2 h after cisplatin treatment in MND1-depleted MCF-7 cells and controls. No significant differences were observed in either repair pathway upon MND1 depletion (Fig. S2E). We directly measured DNA damage repair pathway efficiency using DR-GFP (for HR) and EJ5-GFP (for NHEJ) reporter assays in U2OS cells (a readily transfectable reporter cell line). The results demonstrate that MND1 specifically and substantially promoted HR repair, with no significant effect on NHEJ (Fig. 3G). The activation of key HR mediators, including RAD51 and ATM, was subsequently examined. Western blot analysis of cisplatin-treated MDA-MB-231 cells showed that MND1 overexpression upregulated the protein levels of key DDR and cell cycle factors [27–29], including RAD51, phospho-ATM (Ser1981), and E2F1. However, γ-H2AX (Ser139), as a DNA damage marker, decreases more rapidly after 2 h in the MND1 overexpression group (Fig. 3H). In MND1-depleted MCF-7 cells treated with doxorubicin, western blot analysis of the indicated markers revealed opposite protein kinetic changes at the corresponding time points (Fig. S2F). Collectively, these data demonstrate that MND1 enhances homologous recombination-mediated DNA damage repair, leading to increased chemoresistance in breast cancer cells.

### MND1 binds to RAD51 and inhibits its proteasomal degradation

**Fig. 4:**
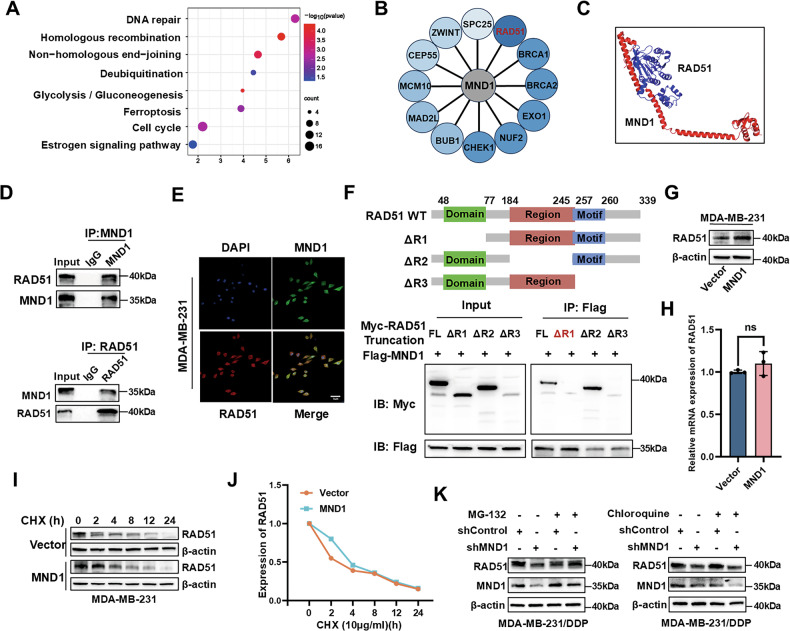
MND1 binds to RAD51 and inhibits its proteasomal degradation. **A** KEGG pathway enrichment analysis of MND1-interacting proteins identified by immunoprecipitation‑mass spectrometry (IP-MS). **B** List of the top 12 high-confidence MND1-binding partners from the IP-MS data. **C** Computational model predicting the direct binding interface between MND1 and RAD51. **D** The endogenous interaction between MND1 and RAD51 in breast cancer cells by co-IP analysis. **E** Immunofluorescence showing nuclear colocalization of endogenous MND1 (green) and RAD51 (red) in MDA-MB-231 cells. Scale bar, μm. **F** Schematic of RAD51 full-length and deletion mutants (top). Co-IP assays in HEK293T cells (bottom) identify the R1 domain of RAD51 as essential for MND1 binding. RAD51 protein and mRNA levels in MND1-overexpressing MDA-MB-231 and control cells determined by western blot (**G**) and RT-qPCR (**H**). **I**, **J** RAD51 expression in MND1-overexpressing MDA-MB-231 and control cells treated with cycloheximide (CHX) for the indicated times, determined by western blot (**I**). Quantification of RAD51 protein levels normalized to β-actin is shown in (**J**). **K** RAD51 protein levels in MND1-depleted MDA-MB-231 and control cells treated with the proteasome inhibitor MG132 (left) or the lysosome inhibitor chloroquine (right), determined by western blot.

To identify MND1-interacting proteins, we performed immunoprecipitation-mass spectrometry (IP-MS) in breast cancer cells. Gene Ontology analysis of the MND1 interactome revealed significant enrichment for pathways involved in DNA damage repair, HR, and cell cycle regulation (Fig. 4A). Analysis of the IP-MS data using the STRING database (https://string-db.org/) prioritized RAD51 as a high-confidence MND1-binding partner and a key cancer-related protein (Fig. 4B). RAD51 is central to DSB repair via the homologous recombination pathway [30, 31]. The potential direct interaction between MND1 and RAD51 was computationally predicted and modeled using protein–protein docking (GRAMM-X, PDBePISA, PyMOL2), with the structural representation rendered in PyMOL (Fig. 4C). We confirmed by co-IP the interaction between endogenous MND1 and RAD51 in MDA-MB-231 cells (Fig. 4D), as well as the interaction between exogenous MND1 and RAD51 in MCF-7 cells (Fig. S3A), a finding that also applies to the two purified human recombinant proteins (Fig. S3B). We further observed their strong colocalization in the nucleus by immunofluorescence (Fig. 4E) in breast cancer cells. To map the domain responsible for binding, we co-expressed full-length and a series of truncated RAD51-Myc mutants with MND1-Flag in HEK293T cells and performed co-IP assays. The results showed that while full-length RAD51, ΔR2, and ΔR3 all bound to MND1, the ΔR1 mutant failed to interact, mapping the critical MND1-binding domain to the R1 region of RAD51 (Fig. 4F). We found that MND1 overexpression increased RAD51 protein levels (Fig. 4G) without altering its mRNA levels in MDA-MB-231 cells (Fig. 4H), suggesting post-translational regulation. This regulation was also validated in MCF-7 cells (Fig. S3C, D). To assess the effect on protein stability, we treated MND1-overexpressing MDA-MB-231 and control cells with cycloheximide (CHX) to block new protein synthesis. MND1 overexpression significantly extended the half-life of RAD51 protein (Fig. 4I, J). To identify the degradation pathway, we treated MND1-depleted MDA-MB-231 and control cells with MG132 (a proteasome inhibitor) or chloroquine (a lysosome inhibitor). MG132, but not chloroquine, effectively rescued the decrease in RAD51 protein expression levels caused by MND1 knockdown (Fig. 4K). Together, these results suggest that MND1 directly binds to RAD51 and stabilizes it by inhibiting ubiquitin-proteasome-mediated degradation.

### MND1 stabilizes RAD51 through USP5-mediated deubiquitination

**Fig. 5:**
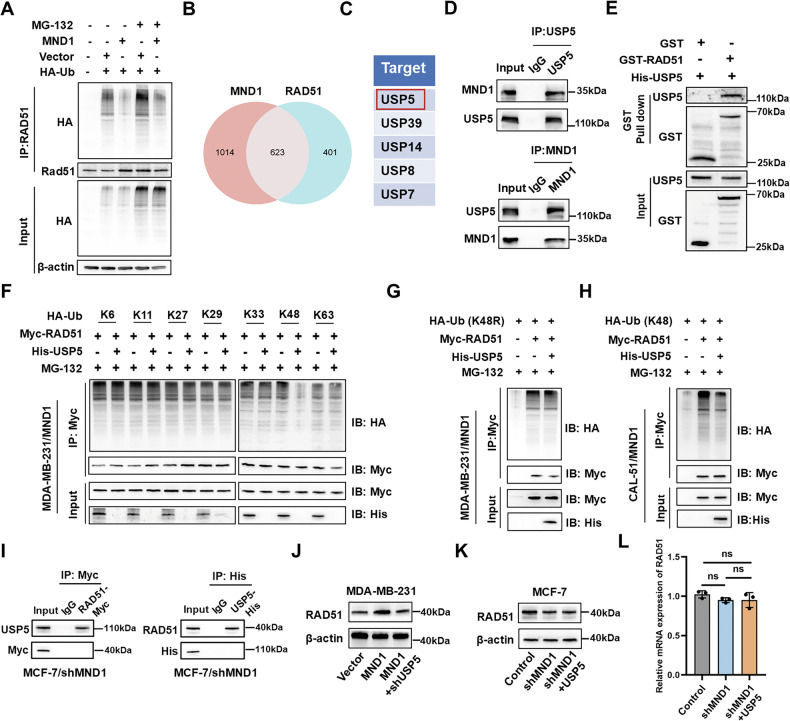
MND1 stabilizes RAD51 through USP5-mediated deubiquitination. **A** The RAD51 polyubiquitination regulated by MND1 in MND1-overexpressing MDA-MB-231 and control cells treated with MG132 determined by co-IP analysis. **B** Venn diagram identifying overlapping interacting partners of MND1 and RAD51 from IP-MS data. **C** Ranking of deubiquitination-related enzymes from the overlapping gene set in (B), identifying USP5 as the top candidate. **D** Interaction between endogenous MND1 and USP5 analyzed by co-IP in MDA-MB-231 cells. **E** GST pull-down assay demonstrating a direct interaction between purified USP5 and RAD51 proteins. **F** USP5-mediated specific polyubiquitination site of RAD51 determined by co-IP analysis. **G**, **H** MND1/USP5 regulates K48‑linked ubiquitination of RAD51 in breast cancer. USP5 fails to deubiquitinate RAD51 when a K48R ubiquitin mutant is expressed in MND1‑overexpressing MDA‑MB‑231 cells (**G**). USP5 suppresses K48‑linked ubiquitination of RAD51 in MND1‑overexpressing CAL‑51 cells (**H**). **I** Interaction between exogenous RAD51 and USP5 analyzed by co-IP in MND1‑depleted MCF‑7 cells. **J** RAD51 expression in vector, MND1‑overexpressing, or MND1‑overexpressing+USP5‑depleted MDA‑MB‑231 cells determined by western blot. RAD51 expression in control, MND1‑depleted, or MND1‑depleted+USP5‑overexpressing MCF‑7 cells determined by western blot (**K**) and RT‑qPCR (**L**).

We next asked whether MND1 regulates RAD51 through the ubiquitin-proteasome pathway. An in vivo ubiquitination assay in MDA-MB-231 cells (treated with MG132 to stabilize ubiquitinated proteins) showed that MND1 overexpression markedly reduced the polyubiquitination of RAD51 (Fig. 5A). Since MND1 itself is not a deubiquitinating enzyme (DUB), we hypothesized that it recruits a DUB to stabilize RAD51. By intersecting the MND1 and RAD51 interactomes from our IP-MS data and ranking the common hits with deubiquitinase activity (Fig. 5B), we identified ubiquitin specific peptidase 5 (USP5) as the leading candidate (Fig. 5C). USP5, a deubiquitinase frequently overexpressed in multiple cancers and linked to poor prognosis [32–34], has been reported to stabilize oncoproteins like Twist1 to promote cancer progression [35]. We confirmed the MND1-USP5 interaction by co-IP (Fig. 5D). Furthermore, a GST pull-down assay showed that USP5 binds directly to RAD51 (Fig. 5E). We next determined the specific ubiquitin linkage involved. Using ubiquitin mutants that primarily form K48-linked chains, we found that USP5 specifically removed K48-linked polyubiquitin chains from RAD51 (Fig. 5F). Consistent with this, USP5 failed to reduce RAD51 ubiquitination when a K48R ubiquitin mutant was expressed, confirming the specificity for K48-linked chains (Fig. 5G). The ability of the MND1-USP5 axis to suppress K48-linked ubiquitination and stabilize RAD51 was consistently observed in CAL-51 cells (Fig. 5H). Interestingly, co‑IP assays showed that neither RAD51 nor USP5 could immunoprecipitate the other in MND1‑knockdown MCF‑7 cells (Fig. 5I). In MDA-MB-231 cells, MND1 overexpression upregulated RAD51 protein levels, whereas concomitant USP5 knockdown attenuated this upregulation (Fig. 5J). In MCF-7 cells, MND1 knockdown reduced RAD51 protein expression; however, simultaneous USP5 overexpression failed to rescue RAD51 protein levels (Fig. 5K). Notably, neither USP5 knockdown nor overexpression significantly affected RAD51 mRNA levels in either cell line (Fig. 5L), indicating that USP5 regulates RAD51 primarily at the post-translational level. Further drug sensitivity assays revealed that USP5 knockdown in MND1-overexpressing MDA-MB-231 cells restored the sensitivity of breast cancer cells to doxorubicin, cisplatin, and olaparib (Fig. S3E). Taken together, these results demonstrate that MND1 recruits USP5 to mediate RAD51 deubiquitination at lysine 48, thereby protecting it from proteasomal degradation.

### E2F1 transactivates MND1 expression in chemoresistant breast cancer cells

**Fig. 6:**
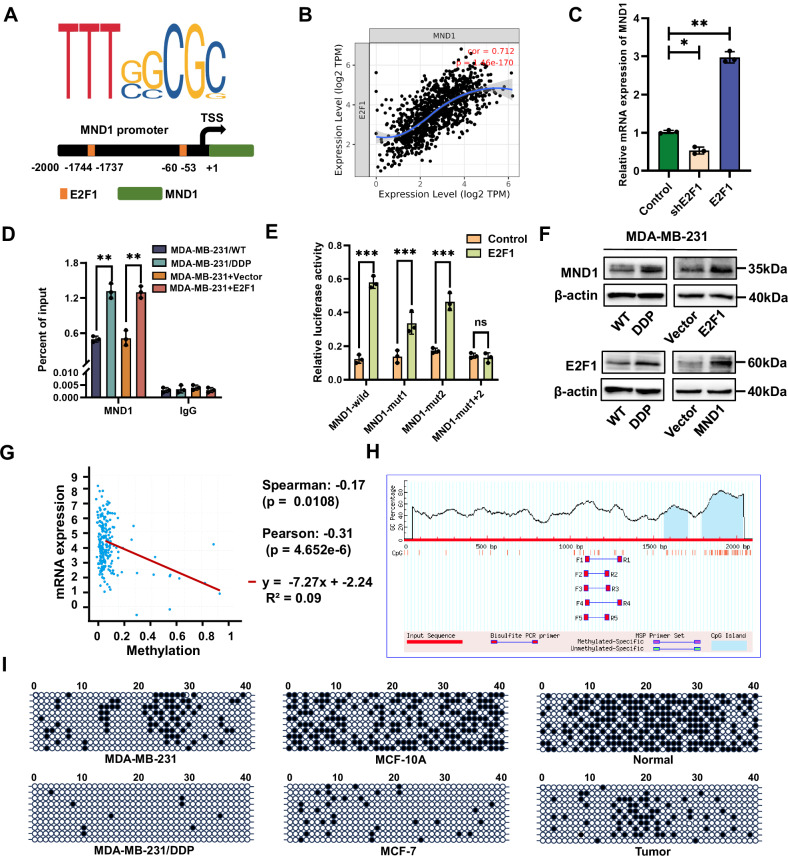
E2F1 transactivates MND1 expression in chemoresistant breast cancer cells. **A** Schematic of the predicted E2F1 binding site within the MND1 promoter region, as identified by UCSC and JASPAR analyses. **B** Correlation analysis of E2F1 and MND1 mRNA expression levels in the TCGA-BRCA cohort. **C** MND1 mRNA levels in MDA-MB-231 cells with modulated MND1 expression, determined by RT‑qPCR. **D** ChIP‑qPCR analysis of E2F1 binding to the MND1 promoter in E2F1‑overexpressing or cisplatin‑resistant MDA‑MB‑231 cells, as well as control cells. **E** MND1 promoter activity in response to E2F1, determined by dual‑luciferase reporter assay. **F** MND1 (upper) and E2F1 (lower) protein levels in the indicated cells, determined by western blot. **G** Correlation between MND1 mRNA expression and its promoter methylation level, analyzed by cBioPortal. **H** Prediction of a CpG island within the MND1 promoter region using MethPrimer. **I** Bisulfite sequencing PCR analysis of MND1 promoter methylation status in normal versus tumor tissues, and in chemotherapy‑sensitive versus chemotherapy‑resistant cell lines. Each row represents a single clone, with filled and open circles indicating methylated and unmethylated CpG sites, respectively. *p < 0.05, **p < 0.01.

To investigate the upstream regulation of MND1 expression, we performed sequence analysis of the MND1 promoter using the UCSC (https://genome.ucsc.edu/) and JASPAR (https://jaspar.genereg.net/) databases. This analysis identified E2F transcription factor 1 (E2F1), a key regulator of the cell cycle, DDR, and a known downstream effector of both MND1 and ATM [36], as a top candidate (Fig. 6A). Analysis of public transcriptomic data revealed a significant positive correlation between E2F1 and MND1 expression (Fig. 6B). We validated this correlation experimentally using RT-qPCR, which showed that modulating E2F1 expression led to corresponding changes in MND1 mRNA expression levels (Fig. 6C). We next assessed whether E2F1 binds to the MND1 promoter using ChIP-qPCR. The results demonstrated enhanced E2F1 binding in cisplatin-resistant MDA-MB-231 cells compared to parental controls. Consistent with this, E2F1 overexpression also increased promoter binding (Fig. 6D). The functional consequence of this binding was confirmed by dual-luciferase reporter assay, which showed that E2F1 binding significantly activated transcription from the MND1 promoter (Fig. 6E). Western blot analysis confirmed that both MND1 and E2F1 protein levels were elevated in MDA-MB-231/DDP cells compared to parental MDA-MB-231 cells (Fig. 6F, left). Furthermore, MND1 and E2F1 mutually regulated each other’s protein levels in both MDA-MB-231 (Fig. 6F, right) and MCF-7 cells (Fig. S3F), suggesting a positive feedback loop. Based on evidence that combined AZA (a DNA methyltransferase inhibitor) and TSA (a histone deacetylase inhibitor) treatment increases chromatin accessibility and gene expression [37–39], we hypothesized that MND1 promoter methylation status modulates its own chromatin accessibility and chemosensitivity. Bioinformatic analysis of the TCGA-BRCA cohort *via* cBioPortal confirmed a significant negative correlation between MND1 mRNA expression and promoter methylation levels (Fig. 6G). We identified a CpG island within the MND1 promoter region using MethPrimer (http://www.urogene.org/methprimer/) (Fig. 6H). BSP directly demonstrated that the MND1 promoter region is hypomethylated in breast cancer tissues and chemoresistant breast cancer cell lines compared to normal tissues and chemosensitive cells (Fig. 6I). To further investigate how E2F1 regulates MND1 methylation, co-IP analysis in MDA-MB-231/DDP cells revealed that E2F1 interacted with the demethylases TET1 and TET2, but did not pull down the methyltransferase DNMT3B (Fig. S3G). Furthermore, knockdown of TET1 and TET2 in E2F1-overexpressing MDA-MB-231 cells led to decreased MND1 mRNA levels (Fig. S3H). These results suggest that E2F1 may cooperate with TET1 and TET2 to demethylate the promoter region of MND1, thereby upregulating MND1 mRNA expression. Taken together, our results establish that E2F1 transactivates MND1 in chemoresistant breast cancer cells, an effect potentiated by promoter hypomethylation.

### MND1 reduces chemosensitivity in vivo and its expression correlates with poor prognosis

**Fig. 7:**
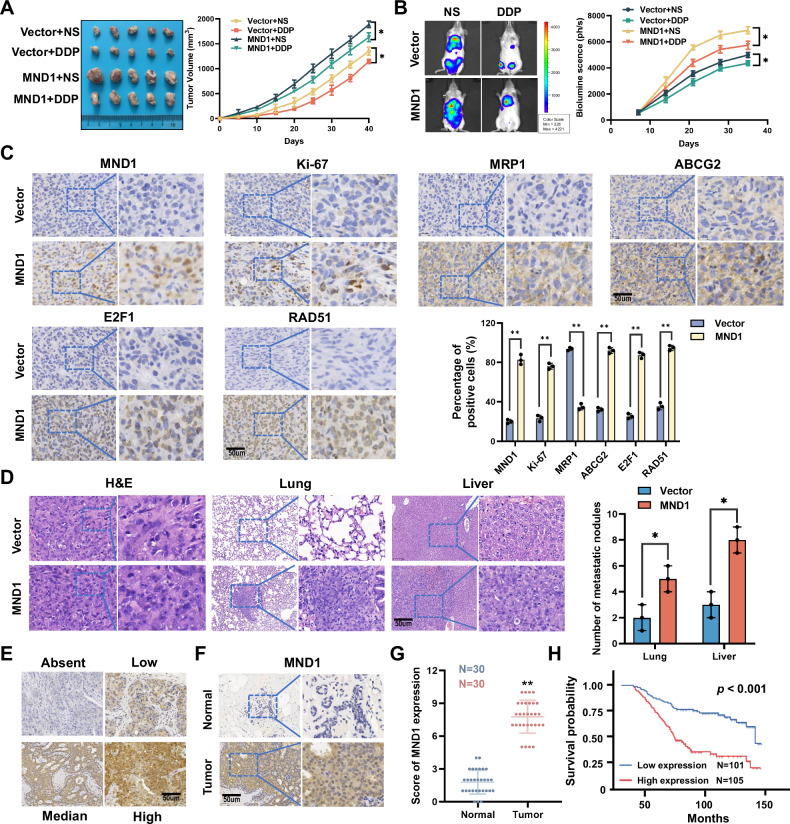
MND1 reduces chemosensitivity in vivo and its expression correlates with poor prognosis. **A** Tumor volume growth curves of orthotopic xenografts derived from MND1-overexpressing MDA-MB-231 cells in SCID mice, treated with vehicle or cisplatin. **B** Metastatic burden assessed by bioluminescence imaging (representative images, left) and quantification of photon flux (right) following intravenous injection of the indicated cells. **C** Representative immunohistochemical (IHC) staining and scoring of proliferation (Ki-67), drug resistance (MRP1, ABCG2), and DNA damage repair (RAD51) and cell cycle progression (E2F1) markers in resected xenograft tumors. **D** Representative H&E staining of lung and liver sections from mice, with arrows indicating metastatic foci. **E** Schematic of the standardized scoring system used for IHC analysis. **F**, **G** MND1 expression in clinical human breast cancer specimens and adjacent normal breast tissue analyzed by IHC. Each dot represents one sample pair. **H** Kaplan-Meier survival analysis comparing patients with high (red) versus low (blue) MND1 expression for overall survival. *p < 0.05, **p < 0.01.

To further evaluate the role of MND1 in breast cancer, we employed two xenograft models in SCID mice: orthotopic mammary fat pad injection of MDA-MB-231 cells to assess primary tumor growth, and intravenous injection to model experimental metastasis. For each model, mice bearing control or MND1-overexpressing tumors were randomized to receive weekly intraperitoneal injections of either vehicle (0.9% saline) or cisplatin (5 mg/kg). MND1 overexpression significantly promoted orthotopic tumor growth and lung/liver metastasis, and markedly reduced the efficacy of cisplatin treatment in both models (Fig. 7A, B). In contrast, knockdown of MND1 significantly suppressed orthotopic tumor growth and improved cisplatin sensitivity (Fig. S3I). IHC analysis of the orthotopic tumors revealed that MND1-overexpressing tumors exhibited significantly higher levels of the proliferation marker Ki-67, the drug efflux transporters MRP1 and ABCG2/BCRP [40–43], cell cycle-related transcription factors E2F1, and the homologous recombination factor RAD51 (Fig. 7C).

H&E staining further confirmed a substantially higher burden of lung and liver metastases in mice injected with MND1-overexpressing cells, even after cisplatin treatment, whereas metastases were minimal in cisplatin-treated control mice (Fig. 7D). We next validated the clinical relevance of MND1 in a cohort of 30 paired breast cancer and adjacent normal tissues by IHC staining, using a standardized scoring system (Fig. 7E). Consistent with our in vitro and xenograft findings, MND1 protein expression was significantly higher in human breast cancer tissues compared to matched normal adjacent tissues (Fig. 7F, G). Critically, survival analysis of an independent cohort of 206 breast cancer patients confirmed that high MND1 expression was significantly associated with poorer overall survival (Fig. 7H). Together, these results demonstrate that MND1 drives tumor growth, metastasis, and chemotherapy resistance, and serves as a potential prognostic marker in patients with breast cancer.

## Discussion

**Fig. 8:**
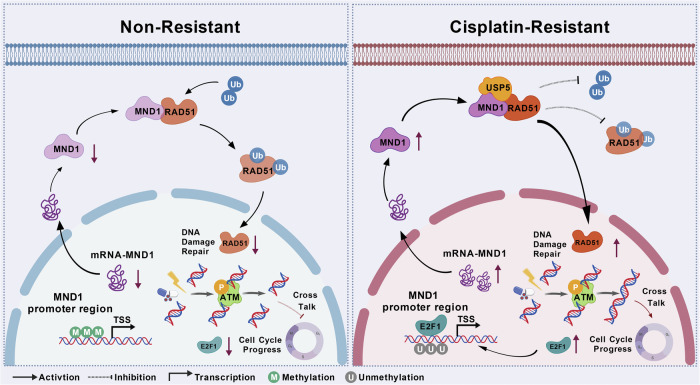
Schematic model depicting MND1’s role in promoting homologous recombination repair and chemoresistance through a positive feedback loop.

Chemoresistance remains a major clinical challenge in breast cancer management, frequently resulting in disease recurrence and metastasis. Here, we identify MND1 as a central driver of this malignant phenotype. We delineate a molecular circuit—the E2F1/MND1/USP5/RAD51 positive feedback loop—that drives MND1-mediated chemoresistance by hyperactivating the DNA damage repair machinery (Fig. 8).

MND1 plays a critical role in breast cancer pathogenesis by promoting aggressive tumor behavior and conferring therapy resistance. Consistent with findings in other cancers, MND1 expression is elevated in breast cancer tissues and correlates with poorer patient prognosis [44]. Functionally, MND1 drives oncogenic processes by enhancing cancer cell proliferation and metastatic potential. This finding is consistent with the established role of oncogenes in promoting tumor growth and invasion. Elevated MND1 levels conferred resistance to multiple chemotherapeutic agents (cisplatin, doxorubicin, olaparib) across various breast cancer models.

MND1 exhibits a distinct expression pattern across breast cancer subtypes. MND1 expression is higher in luminal breast cancer cells (e.g., MCF-7, T47D) than in normal breast epithelial cells (MCF-10A) or TNBC cells (e.g., MDA-MB-231, CAL-51, BT-549). This pattern suggests that MND1’s oncogenic role is context-dependent and influenced by the distinct molecular landscapes of different breast cancer subtypes. The pronounced heterogeneity and aggressive nature of TNBC, which lacks estrogen receptor (ER), progesterone receptor (PR), and human epidermal growth factor receptor 2 (HER2) expression, may contribute to this differential MND1 expression [45]. The ER-positive nature of luminal subtypes may create a regulatory environment conducive to MND1 expression. For example, estrogen signaling or other luminal-specific transcription factors may positively regulate MND1 expression. Conversely, the low MND1 expression in TNBC cells may reflect their distinct cellular origin or repressive regulatory mechanisms, such as promoter hypermethylation or specific microRNA profiles, characteristic of the basal-like/TNBC phenotype [46]. Furthermore, the low MND1 expression in MCF-10A cells may reflect their non-transformed state, where MND1 is not required [9]. The ability of MND1 to promote DNA damage repair and its differential expression among breast cancer molecular subtypes may contribute to the subtype-specific chemosensitivity observed in our study. Among the major molecular subtypes of breast cancer, TNBC is generally the most sensitive to DNA-damaging chemotherapeutic agents such as doxorubicin, cisplatin and olaparib, due to its intrinsic defects in homologous recombination repair such as BRCA1/2 mutations [47, 48]. In contrast, luminal breast cancer, characterized by slow proliferation and robust DNA repair capacity, is typically less chemosensitive [49]. HER2-positive breast cancer exhibits an intermediate sensitivity, but its response is markedly improved by the addition of anti-HER2 targeted therapy [50].

We elucidated the precise molecular mechanism by which MND1 confers resistance to DNA-damaging therapies. RAD51 contributes to cisplatin resistance in breast cancer through multiple mechanisms. CtBP1 transactivates RAD51 to confer cisplatin resistance [51]. Additionally, Jab1 regulates RAD51 in a p53-dependent manner, and RAD51 overexpression restores chemoresistance in Jab1-deficient cells [52]. Zelceski et al. demonstrated the colocalization of MND1 with RAD51, while Koob et al. highlighted the importance of MND1-driven homologous recombination (HR) for radiosensitivity [53]. We demonstrated that MND1 physically interacts with RAD51, a crucial recombinase for homologous recombination (HR)-mediated repair of DNA double-strand breaks [54]. MND1 acts as a scaffold to recruit the deubiquitinase USP5. USP5 specifically removes K48-linked polyubiquitin chains from RAD51 [55, 56]. By removing K48-linked ubiquitin chains—a signal for proteasomal degradation—the MND1-USP5 complex stabilizes RAD51 [57]. This stabilization increases the intracellular RAD51 pool, facilitating the formation of RAD51 nucleoprotein filaments required for efficient HR [58]. Consequently, this enhanced DNA repair capacity promotes breast cancer cell survival under genotoxic stress from chemotherapy and radiotherapy, leading to treatment resistance. Pan-cancer analysis has revealed that MND1 can interact with BRCA2 [12], suggesting a potential functional association between MND1 and BRCA2 in HR repair. Meanwhile, BRCA2 is a key HR factor that mediates HR by recruiting RAD51 to DNA double-strand break (DSB) sites and facilitating the formation of RAD51 nucleoprotein filaments [59], which is essential for HR repair progression. As a scaffold protein, PALB2 can connect BRCA1 and BRCA2, stabilize the RAD51 filament, and promote the assembly of the HR complex [60]. Combined with our IP-MS finding that MND1 interacts with BRCA2, we propose that MND1 may also function as a scaffold protein to facilitate the assembly of the HR complex (including MND1, RAD51, BRCA2, and potentially PALB2).

We further elucidated the upstream regulatory mechanisms driving high MND1 expression in chemoresistant breast cancer. The transcription factor E2F1, a key regulator of cell cycle progression and the DNA damage response, directly binds the MND1 promoter and acts as a potent transcriptional activator. E2F1 is overexpressed in breast cancer and associated with aggressive tumor behavior and poor clinical outcomes [61, 62]. For example, E2F1 and EIF4A3 promote TNBC carcinogenesis through the circSEPT9/miR-637/LIF axis [63]. Additionally, lncRNA AGPG blocks PURα/E2F1 complex formation, activating E2F1 and promoting endocrine resistance [36]. In response to genotoxic stress, E2F1 is transcriptionally induced and post-translationally modified through phosphorylation by ATM/ATR and CHK2 kinases, which stabilizes the protein and enhances its activity. These modifications increase E2F1 levels and facilitate its recruitment to DNA damage sites, promoting HR-mediated repair [64, 65]. We demonstrated that E2F1 is regulated by MND1, which also binds the MND1 promoter and regulates its expression, establishing a positive feedback loop. Thus, MND1 is a novel E2F1 target gene, directly linking this oncogenic pathway to DNA repair machinery. It has been reported that KLF6 binds to the E2F1 promoter and transcriptionally represses E2F1 under low MND1 conditions, while MND1 overexpression competitively interacts with KLF6 to release E2F1 from transcriptional suppression [11]. Building on this basis, our findings further revealed that E2F1 recruits and cooperates with TET1/TET2 to target the MND1 promoter in chemoresistant breast cancer cells. TET family enzymes catalyze the oxidation of 5mC to 5hmC, thereby reducing MND1 promoter methylation, upregulating MND1 transcription, and ultimately activating a positive feedback regulatory loop [66].

In this study, we observed distinct dynamic patterns of DNA damage and repair proteins. As shown in Fig. 3H, p-ATM/γ-H2AX and RAD51 displayed biphasic alterations in both groups with different temporal kinetics. In MND1-overexpressing cells, p-ATM and γ-H2AX increased within 2 h and declined at 8 h, while these damage markers kept rising until 8 h in control cells and decreased at 16 h, which was in line with previous reports. Notably, RAD51 showed opposite trends: MND1 overexpression sustained RAD51 upregulation until 8 h, followed by a decline at 16 h, whereas RAD51 in control cells peaked at 2 h and gradually decreased thereafter. Mechanistically, cisplatin-induced DNA damage promotes MND1-mediated deubiquitination of RAD51, thereby maintaining its high level. As an early DNA damage sensor, p-ATM initiates damage response, and sustained RAD51 facilitates efficient homologous recombination. Rapid DNA damage resolution in MND1-overexpressing cells accelerates the decline of p-ATM and γ-H2AX, resulting in temporal dissociation between damage and repair indicators.

Existing studies have validated that ATM-mediated E2F1 phosphorylation inhibits its ubiquitin-dependent degradation and enhances its stability, while ATM-dependent E2F1 accumulation in the nucleolus is a characteristic feature of nucleolar stress in the early response to DNA damage [67, 68]. To explore the role of ATM in this regulatory axis, CAL-51 cells were treated with cisplatin combined with DMSO or the ATM inhibitor KU55933. Compared with the DMSO group, KU55933 markedly reduced p-ATM levels and downregulated its downstream molecules E2F1, MND1 and RAD51 (Fig. S4A). Meanwhile, ATM inhibition suppressed MND1 mRNA transcription (Fig. S4B). Comet assays confirmed sustained DNA damage and impaired repair efficiency in KU55933-treated cells (Fig. S4C). Immunofluorescence further verified that KU55933 blocked DNA damage-induced RAD51 foci formation (Fig. S4D). Moreover, ATM inhibition significantly sensitized MDA-MB-231/DDP and MCF-7 cells to cisplatin, doxorubicin and olaparib (Fig. S4E–H). Collectively, blocking ATM activity aggravates chemotherapy-induced DNA damage and enhances breast cancer chemosensitivity by inhibiting the E2F1-MND1-RAD51 axis.

Building on this evidence, we investigated the E2F1/MND1/USP5/RAD51 positive feedback loop in breast cancer. Chemoresistance remains a major challenge, particularly in TNBC, which relies heavily on chemotherapy. We found that chemosensitivity correlates with MND1 promoter methylation. Low methylation—common in certain chemoresistant TNBC and luminal subtypes—enables E2F1-driven MND1 expression. Elevated MND1 promotes proliferation, EMT, drug efflux, and recruits USP5 to deubiquitinate and stabilize RAD51, thereby enhancing homologous recombination repair after DNA damage. Efficient repair sustains genomic stability and cell cycle progression, generating more E2F1, which further activates MND1 transcription, forming a self-reinforcing loop that drives chemoresistance. Conversely, high MND1 promoter methylation—seen in chemosensitive TNBC and normal tissues—keeps MND1 low and prevents this resistance circuit.

Our study has several limitations. Deubiquitination primarily occurs in the cytoplasm, apart from its roles involving nuclear histones, transcription factors, proteins that maintain nuclear ubiquitin stability [69]. Our findings demonstrate that MND1 recruits USP5 to deubiquitinate RAD51, a process that likely occurs in the cytoplasm. However, the mechanism of RAD51 nuclear translocation following its stabilization remains unclear. For instance, it is unknown whether specific nuclear localization signals or transcription factors facilitate this translocation. Epigenetic regulation plays a pivotal role in gene expression [70, 71]. For example, specific CpG sites in the CD155 promoter may serve as biomarkers for breast cancer surveillance [72]. We hypothesized that differential methylation of the MND1 promoter affects chromatin accessibility, thereby regulating E2F1 binding and chemosensitivity. We found that E2F1 may mediate demethylation of the MND1 promoter region by regulating the demethylases TET1 and TET2, although the detailed regulatory mechanism remains to be investigated. Future studies should characterize chromatin accessibility at the MND1 promoter using high-resolution methods such as ATAC-seq or DNase-seq.

In conclusion, we propose a model wherein promoter hypomethylation and E2F1-mediated transcription drive MND1 overexpression, leading to USP5-dependent RAD51 stabilization and hyperactive HR repair. This cycle is perpetuated by reciprocal reinforcement between MND1 and E2F1. Targeting this axis by disrupting the MND1-RAD51 interaction or inhibiting USP5 activity represents a novel strategy to overcome breast cancer chemoresistance. As MND1 is largely dispensable in somatic cells, it is an attractive therapeutic target for future drug development.

## Supplementary information

Original blot images

Supplementary materials

## Data Availability

All the data generated or analyzed during the course of this study have been fully documented and are presented in this article and its supplementary files.
