# Supplementary material for: MND1 reduces breast cancer chemosensitivity by promoting RAD51-mediated homologous recombination repair
Source: Cell Death Dis. 2026 Jun 3;17(1):699. doi: 10.1038/s41419-026-08912-w (PMC13448082; doi:10.1038/s41419-026-08912-w)
Supplement: Supplementary file 1 [file 41419_2026_8912_MOESM1_ESM.docx]

Figure 2B

MND1 β-actin

Figure 2C

MND1(left) MND1(right)

β-actin(left) β-actin(right)

Figure 2K

E-cadherin (left) E-cadherin (right)

N-cadherin (left) N-cadherin (right)

ABCG2 (left) ABCG2 (right)

β-actin (left) β-actin (right)

Figure 3H

p-ATM (left) p-ATM (right)

γ-H2AX (left) γ-H2AX (right)

RAD51 (left) RAD51 (right)

E2F1 (left) E2F1 (right)

β-actin (left) β-actin (right)

Figure 4D

RAD51 (up) MND1 (up)

MND1 (down) RAD51 (down)

Figure 4F

Myc (left) Myc (right)

Flag (left) Flag (right)

Figure 4G

RAD51 β-actin

Figure 4I

RAD51 (up) β-actin (up)

RAD51 (down) β-actin (down)

Figure 4K

RAD51 (left) RAD51 (right)

MND1 (left) MND1 (right)

β-actin (left) β-actin (right)

Figure 5A

HA (up) HA (down)

RAD51 β-actin

Figure 5D

MND1 (up) USP5 (up)

USP5 (down) MND1 (down)

Figure 5E

USP5 (up) USP5 (down)

GST (up) GST (down)

Figure 5F

HA (left) HA (left)

Myc (left) (up) Myc (left) (down)

Myc (right) (up) Myc (right) (down)

His (left) His (right)

Figure 5G

HA His

Myc (up) Myc (down)

Figure 5H

HA His

Myc (up) Myc (down)

Figure 5I

USP5 Myc

RAD51 His

Figure 5J

RAD51 β-actin

Figure 5K

RAD51 β-actin

Figure 6F

MND1 (left) MND1 (right)

β-actin (up) (left) β-actin (up) (right)

E2F1 (left) E2F1 (right)

β-actin (down) (left) β-actin (down) (right)

Figure S1A

MND1 β-actin

Figure S2E

XPA MLH1

XPC MSH2

ERCC1 MSH6

β-actin (left) β-actin (right)

Figure S2F

p-ATM (Ser1981) (left) p-ATM (Ser1981) (right)

γ-H2AX (Ser139) (left) γ-H2AX (Ser139) (right)

RAD51 (left) RAD51 (right)

E2F1 (left) E2F1 (right)

β-actin (left) β-actin (right)

Figure S3A

RAD51 Flag

MND1 Myc

Figure S3B

MND1 RAD51

Figure S3C

RAD51 β-actin

Figure S3F

MND1 E2F1 β-actin

Figure S3G

E2F1 TET1

TET2 DNMT3B

Figure S4A

p-ATM E2F1

MND1 RAD51 β-actin
