# Supplementary material for: MND1 reduces breast cancer chemosensitivity by promoting RAD51-mediated homologous recombination repair
Source: Cell Death Dis. 2026 Jun 3;17(1):699. doi: 10.1038/s41419-026-08912-w (PMC13448082; doi:10.1038/s41419-026-08912-w)
Supplement: Supplementary file 2 [file 41419_2026_8912_MOESM2_ESM.docx]

**Supplementary Material**

**Figure S1.** **MND1 promotes breast cancer proliferation, migration, invasion and chemoresistance *in vitro*.**

**A and B,** Stable MND1-overexpressing CAL-51 cell line was validated by western blot (A) and RT-qPCR (B).

**C–E,** Functional consequences of MND1 modulation on cell proliferation were assessed by CCK-8 (C), colony formation (D), and EdU (E) assays.

**F and G,** Effects of MND1 on cell migration and invasion were evaluated by wound healing (F) and Transwell (G) assays.

**H–J,** Dose-response curves and calculated IC₅₀ values for doxorubicin, cisplatin, and olaparib in CAL-51 (H), MDA-MB-231 (I), and MCF-7 cells (J) with modulated MND1 expression. **p* < 0.05, ***p* < 0.01.

**Figure S2. MND1 enhances homologous recombination repair and confers chemoresistance.**

**A,** Quantification of DNA damage (comet tail moment) by neutral comet assay at indicated time points after chemotherapy.

**B,** Immunofluorescence analysis of γ-H2AX foci formation and resolution over time post-chemotherapy, indicating DNA damage repair kinetics.

**C and D,** Flow cytometry analysis of cell cycle distribution (C) and apoptosis (D) in MND1-overexpressing CAL-51 and control cells with or without chemotherapy.

**E,** Western blot analysis of key NER (XPA, XPC, ERCC1) and MMR (MLH1, MSH2, MSH6) proteins in MND1-depleted MCF-7 and control cells 2 hours after cisplatin treatment.

**F,** Western blot analysis of key DNA damage response (phospho-ATM, γ-H2AX, RAD51) and cell cycle (E2F1) proteins in MND1-depleted MCF-7 and control cells treated with doxorubicin. **p* < 0.05, ***p* < 0.01, ****p* < 0.001.

**Figure S3. Supporting data related to the E2F1/MND1/USP5/RAD51 regulatory loop.**

**A,** The interaction between exogenous MND1 and RAD51 analyzed by co-IP in MCF-7 cells.

**B,** Co-IP analysis of the interaction between human recombinant MND1 and RAD51.

**C and D,** RAD51 protein and mRNA levels in MND1‑depleted MCF-7 and control cells determined by western blot (C) and RT‑qPCR (D).

**E,** Dose–response curves and calculated IC₅₀ values of doxorubicin, cisplatin, and olaparib in MDA-MB-231 cells with vector control, MND1 overexpression, or MND1 overexpression combined with USP5 knockdown.

**F,** MND1 and E2F1 expression in MND1‑ or USP5‑depleted MCF-7 cells determined by western blot.

**G,** Co-IP analysis of E2F1 with demethylases (TET1, TET2) and methyltransferase (DNMT3B).

**H,** MND1 mRNA levels in E2F1‑overexpressing MDA‑MB‑231 cells with TET1 and TET2 depletion, determined by RT‑qPCR.

**I,** Tumor volume growth curves of orthotopic xenografts derived from MND1-depleted MCF-7 cells in SCID mice, treated with cisplatin. **p* < 0.05, ***p* < 0.01, ****p* < 0.001.

**Figure S4. ATM inhibitor sensitizes breast cancer cells to chemotherapy.**

**A,** The expression of p-ATM, E2F1, MND1, and RAD51 in CAL-51 cells untreated, treated with DDP+DMSO, or treated with DDP+KU55933 was determined by western blot.

**B,** The expression of MND1 in CAL-51 cells untreated, treated with DDP+DMSO, or treated with DDP+KU55933 was determined by RT-qPCR.

**C,** Quantification of DNA damage (comet tail moment) by neutral comet assay at indicated time points after chemotherapy.

**D,** Immunofluorescence analysis of RAD51 foci formation and resolution over time post-chemotherapy.

**E,** Dose-response curves and calculated IC₅₀ values for cisplatin in MDA-MB-231/DDP cells under negative control, DMSO, or KU55933 conditions.

**F–H,** Dose-response curves and calculated IC₅₀ values for doxorubicin (F), cisplatin (G), and olaparib (H) in MCF-7 cells under negative control, DMSO, or KU55933 conditions. **p* < 0.05, ***p* < 0.01, ****p* < 0.001.

**Table S1. Oligonucleotides of PCR, shRNAs and siRNAs.**

| **Name** | **Sequence (5’ to 3’)** |
| --- | --- |
| MND1 (F)  MND1 (R) | ATGTCAAAGAAAAAAGGACTGAGTGC  CTTTATTTGCTTGGCCGTTTCA |
| RAD51 (F)  RAD51 (R) | ATGGCAATGCAGATGCAGC  GTCTTTGGCATCTCCCACTCC |
| ΔR1 (F)  ΔR1 (R) | GCAGCTAAATTAGTTCCAATGGG  GTCTTTGGCATCTCCCACTCC |
| ΔR2 (F)  ΔR2 (R)  ΔR2 (F)  ΔR2 (R) | ATGGCAATGCAGATGCAGC  ACTGCCAGAGAGACCATACCTCTC  TTGGCCAGGTTTCTGCGG  GTCTTTGGCATCTCCCACTCC |
| ΔR3 (F)  ΔR3 (R) | ATGGCAATGCAGATGCAGC  ATCAGCGAGTCGCAGAAGCA |
| USP5 (F)  USP5 (R) | ATGGCGGAGCTGAGTGAGG  GCTGGCCACTCTCTGGTAGAAG |
| E2F1 (F)  E2F1 (R) | ATGGCCTTGGCCGGGGCC  GAAATCCAGGGGGGTGAGG |
| Promoter MND1 (F) MND1 (R) | CAGGAGGCTGAGGCAAGAGA  ACGCCGCGGGGGCGGAGC |
| shMND1  shMND1 (anti) | GCTGCTAACAGATGGACTGAT  ATCAGTCCATCTGTTAGCAGC |
| shUSP5  shUSP5 (anti) | CTTTGCCTTCATTAGTCACAT  ATGTGACTAATGAAGGCAAAG |
| shE2F1  shE2F1 (anti) | TAACTGCACTTTCGGCCCTTT  AAAGGGCCGAAAGTGCAGTTA |
| siControl  siControl (anti) | UUCUCCGAACGUGUCACGU  ACGUGACACGUUCGGAGAA |
| siTET1-1  siTET1-1 (anti) | UCGUAGUUGGCUGUUUUUCUU  GAAAAACAGCCAACUACGAAA |
| siTET1-2  siTET1-2 (anti) | AAUUUUCCAGGGCUUAAAGUC CUUUAAGCCCUGGAAAAUUAA |
| siTET1-3  siTET1-3 (anti) | UUAAUUGCUUUAAUUUUCCAG GGAAAAUUAAAGCAAUUAAUU |
| siTET2-1  siTET2-1 (anti) | AUAAUAACUUUUGAAAGAGUG CUCUUUCAAAAGUUAUUAUGG |
| siTET2-2  siTET2-2 (anti) | UAUUCCAUAAUAACUUUUGAA  CAAAAGUUAUUAUGGAAUACC |
| siTET2-3  siTET2-3 (anti) | UACUUUCUUGUGUAAAGUCAG GACUUUACACAAGAAAGUAGA |

**Table S2. Oligonucleotides used for RT-qPCR.**

| **Name** | **Sequence (5’ to 3’)** |
| --- | --- |
| MND1 (F)  MND1 (R) | TGTGAGAGGATCGGAACTTCT CACATCGGCCAATTTTAGCTTTC |
| RAD51 (F)  RAD51 (R) | CAACCCATTTCACGGTTAGAGC TTCTTTGGCGCATAGGCAACA |
| GAPDH (F)  GAPDH (R) | CAAGGTCATCCATGACAACTTTG  GTCCACCACCCTGTTGCTGTAG |

**Table S3. Oligonucleotides used for ChIP and methylation-specific PCR.**

| **Name** | **Sequence (5’ to 3’)** |
| --- | --- |
| MND1 site 1 (F)  MND1 site 1 (R) | CTGGGCAAAAGAGCGAAAC  TGTCACAGCAGCTCTCAGTG |
| MND1 site 2 (F)  MND1 site 2 (R) | AAAGCTGGAAAGCAGTGGGT  GACAGGACAAGTCCGGAAGG |
| BSP (F)  BSP (R) | AAAATTTAAGGGAAAAATAAATAGAT TTACCTTTACTTTTACTACRTTTTCT |

**Data S1**

**Antibodies and reagents**

MND1 Polyclonal antibody (Cat No. 11636-1-AP, rabbit), RAD51 Polyclonal antibody (Cat No. 14961-1-AP, rabbit), USP5 Polyclonal antibody (Cat No. 10473-1-AP, rabbit), MSH6 Polyclonal antibody (Cat No. 18120-1-AP), TET2 Polyclonal antibody (Cat No. 21207-1-AP), BCRP/ABCG2 (Cat No. 27286-1-AP, rabbit) and Beta Actin Monoclonal antibody (Cat No. 66009-1-Ig, mouse) were purchased from Proteintech (China). E2F1 (PT0370R) PT™ Rabbit mAb (YM8222), Ki-67 (PT0321R) PT™ Rabbit mAb (YM8189), MRP1 (PT0945R) PT™ Rabbit mAb (YM8734), XPA Rabbit pAb (YT4914), XPC (PT1464R) PT™ Rabbit mAb (YM9306), ERCC1 (PT1303R) PT™ Rabbit mAb (YM9145), MLH1 (ABT018) Mouse mAb (YM6231), MSH2 (ABT022) Mouse mAb (YM6232), TET1 Rabbit pAb (YT8061) and DNMT3B Rabbit pAb (YT1388) were purchased from ImmunoWay (USA). N-Cadherin (D4R1H) Rabbit Monoclonal Antibody (#13116), E-Cadherin (24E10) Rabbit Monoclonal Antibody (#3195), TWIST1 (E5G9Y) Rabbit Monoclonal Antibody (#90445), Phospho-ATM (Ser1981) (D6H9) Rabbit Monoclonal Antibody (#5883), Phospho-Histone H2A.X (Ser139) (20E3) Rabbit Monoclonal Antibody (#9718), Myc-Tag (9B11) Mouse Monoclonal Antibody (#2276), DYKDDDDK Tag (D6W5B) Rabbit Monoclonal Antibody (#14793), HA-Tag (C29F4) Rabbit Monoclonal Antibody (#3724), His-Tag Antibody (#2365, rabbit) were purchased from Cell Signaling Technology (USA). Recombinant Human RAD51 protein (P1456) was purchased from FineTest (China). Recombinant human MND1 protein (550229) was purchased from NovoPro (China). Doxorubicin (Cat: ID0380), Cisplatin (Cat: IC0440) and Olaparib (Cat: IO0330) were purchased from Solarbio (China). Cycloheximide (HY-12320), MG-132 (HY-13259), Chloroquine (HY-17589A) and KU-55933 (HY-12016) were purchased from MedChemExpress (USA).

**Plasmids, small interfering RNA (siRNA) and transfection**

The following plasmid vectors were used: PCDH-CMV-3×Flag, PCDH-CMV-MCS-3×His, PCDH-CMV-MCS-Myc, pLV3-CMV-MCS-Fluc, and pLKO.1. The HA-Ub plasmid, ubiquitination site mutant plasmids (K6, K11, K27, K29, K33, K48, K63, K6R, K11R, K27R, K29R, K33R, K48R, K63R), DR-GFP, EJ5-GFP, pGL3-Basic, pRL-TK, pGEX-4T-1-RAD51-GST, and pET-30a(+)-USP5-His were purchased from RiboBio (Shanghai, China). The siControl, siTET1-1, siTET1-2, siTET1-3, siTET2-1, siTET2-2, and siTET2-3 were also purchased from RiboBio. All constructs were confirmed by sequencing. For transient transfection, shRNAs and siRNAs were transfected into different cell lines using FuGENE HD Transfection Reagent (Promega, Madison, WI, USA), and plasmids were transfected using TransFast Transfection Reagent (Promega) according to the manufacturers' recommendations. To generate stable cells, lentiviruses (RiboBio, Shanghai, China) were used to infect the corresponding cell lines according to the manufacturer's recommendations.

**Proliferation and invasion assay**

For the CCK‑8 assay, 24 h after transfection, cells were seeded into 96‑well plates at a density of 5 × 10⁴ cells per well. After incubation for the indicated time, cells were incubated with 95 μL of medium and 5 μL of CCK‑8 solution per well at 37°C for 2 h. The medium was then removed, and the precipitated contents were dissolved in 150 μL DMSO. The absorbance at 450 nm was detected using a microplate auto‑reader (Bio‑Rad, Richmond, CA, USA). The EdU assay was conducted using an EdU Assay Kit (RiboBio, China) according to the manufacturer’s instructions. The EdU‑positive cell ratio was counted and analyzed using a fluorescence microscope (Zeiss, Germany).

For the plate colony formation assay, 24 h after transfection, cells were seeded into 6‑well plates at a density of 500 cells per well. After approximately 3 weeks, the colonies were washed with phosphate‑buffered saline (PBS), fixed with 4% paraformaldehyde for 15 minutes at room temperature, washed again with PBS, and then stained with 0.1% crystal violet solution. The number of colonies was counted and compared with the control.

The invasive ability of breast cancer cells in vitro was evaluated using Matrigel‑coated Transwell inserts (BD Biosciences, San Diego, CA, USA). Briefly, 5 × 10⁴ cells in 500 μL serum‑free medium were added to the upper chamber, and medium containing 20% FBS was added to the lower chamber. Twenty‑four hours later, the migrated cells attached to the lower surface were fixed with 4% paraformaldehyde and stained using a three‑step staining method. The membranes were then cut and mounted onto slides with the cells facing upward.

A wound‑healing assay was used to evaluate cell migration. Cells were cultured in six‑well plates. When 70% confluency was reached, the cells were starved overnight, and a wound was scratched in the center of the cell monolayer using a sterile plastic pipette tip. Debris was removed by washing with PBS. Images were captured at 0 h, 12 h, and 24 h after wounding.
